# Why Police Pull Back? Organizational Injustice and Depolicing

**DOI:** 10.1177/10986111261432979

**Published:** 2026-03-10

**Authors:** Gabriel Béliveau, Massimiliano Mulone, Maude Pérusse-Roy

**Affiliations:** 1 141640 Université de Montréal, Montreal, QC, Canada

**Keywords:** depolicing, organizational justice, police oversight, internal investigations

## Abstract

Depolicing refers to a perceived decline in police engagement, particularly in proactive enforcement. Although widely invoked in political and media debates, the concept remains theoretically and empirically contested. Existing research suggests that overall police activity remains relatively stable and that only a minority of officers report reducing their own interventions, raising questions about the origins of disengagement narratives. Drawing on an organizational justice framework, this study analyzes 59 in-depth interviews with Quebec police officers to examine how officers construct accounts of disengagement through lived experiences of oversight. We identify three interrelated dynamics associated with radical disengagement: employer-initiated internal investigations that constrain blame externalization and disrupt occupational solidarity; precautionary administrative measures that produce stigmatization and isolation; and disciplinary responses to morally framed proactive practices, often interpreted as noble cause corruption. We argue that radical depolicing is rooted less in external scrutiny than in perceived organizational injustice.

## Overview

Since the protests in Ferguson, Missouri, following the killing of Michael Brown (Mac Donald, 2016), discussions surrounding police disengagement—“depolicing”—have increased in Western societies (Pomper, 2018). Disengagement, which is both multifaceted and often misunderstood, is defined in different ways. Some officers describe it in psychological terms, as feelings of demotivation or discouragement, while others refer to observable behavioral shifts such as avoidance, hesitation, or reduced initiative (Faubert, 2021). A dominant narrative among the police is that public criticism and heightened scrutiny of the police have contributed to a marked decline in proactive policing (Brown, 2019). Empirical investigations into this issue have increased in the United States during the past decade, spurred in part by the murder of George Floyd and broader disruptions caused by the COVID-19 pandemic (Copeland et al., 2022). Many officers express concern over rising levels of violent crime and have suggested that increased public demands and negative perceptions of the police have impeded operational effectiveness. Such concerns have been widely disseminated, prompting statements from political figures, including former President Barack Obama (Kearns, 2015), as well as sustained media coverage in major news outlets.^1^

Despite sustained attention to depolicing, both the empirical literature and political discourse reveal two persistent tensions. First, decades of research indicate that overall police intervention rates tend to remain relatively stable over time (Copeland et al., 2022). Second, although officers frequently portray depolicing as widespread, only a minority report having substantially reduced their own intervention practices (Brown, 2019; Faubert, 2021). To better understand this gap between collective narratives and reported practices, this article examines how disengagement narratives are constructed and sustained, particularly in relation to officers’ experiences with complaints, oversight mechanisms, and media exposure.

In a recent study (Béliveau et al., 2024), we explored the varying degrees of disengagement discourses among officers and found that a significant subset of those in our sample reported having engaged in severe and socially problematic forms of disengagement. However, our findings at that time did not explain why some officers described themselves as disengaging while others affirmed their continued professional commitment despite similar experiences. Drawing on an organizational justice framework, this article addresses that gap by investigating how officers' experiences with complaints—and, more broadly, with internal and external oversight systems—are linked to differing levels of disengagement narratives, tracing the origins of radical depolicing through a comparative analysis of officer accounts. By identifying key events—from the initial filing of a complaint to the resolution of proceedings—that are perceived as unjust or inequitable, we explore how particular types of experiences may give rise to a subjective sense of injustice that acts as a catalyst for radical forms of professional self-reported withdrawal.

### Evidence on Stability and Change in Police Activity

Research on variations in police interventions—and, by extension, on the presence and scope of depolicing—has produced few consistent or conclusive findings (Bollinger, 2018; Campbell, 2024; Chanin & Sheats, 2018; Copeland et al., 2022; Foster, 2021; Larson et al., 2023; Maguire et al., 2017; Noray, 2024; Pyrooz et al., 2016; Rosenfeld & Wallman, 2019; Shjarback et al., 2017; Towers & White, 2017). Most studies do not identify a substantial decline in police activity or a strong overall effect of disengagement. Only a limited subset points to possible impacts in specific operational domains, such as changes in officers’ field behavior (Kim, 2024; Phillips, 2020; Piza & Connealy, 2022; Powell, 2022; Rushin & Edwards, 2017). The generalizability and methodological robustness of this latter group, however, remain limited. Many focus on isolated practices that are weak indicators of systemic trends (Kim, 2024), are restricted to single cities (Powell, 2022) or departments (Piza & Connealy, 2022), and rarely draw on long-term, multi-site data (Rushin & Edwards, 2017). Cross-regional comparisons also often overlook intra-jurisdictional variation in policing, thereby introducing endogeneity biases and blurring distinctions between cause and effect (Nix et al., 2024).

By contrast, studies reporting no significant changes in police interventions generally rely on broader geographic coverage, such as nationwide analyses in the United States (Pyrooz et al., 2016; Rosenfeld & Wallman, 2019), and on longitudinal data (Chanin & Sheats, 2018; Nix et al., 2024; Noray, 2024). These works examine a wide range of police activities, including stops, detentions, arrests, searches (Shjarback et al., 2017), use of force (Koslicki, 2022), and foot patrols (Marier & Fridell, 2020), and assess their relationship to multiple outcomes. Taken together, this body of research provides little evidence of a widespread or sustained decline in proactive policing consistent with a generalized depolicing phenomenon in the United States (Chanin & Sheats, 2018; Wallace et al., 2018). It also suggests that variations in police activity have limited influence on overall crime trends, casting doubt on claims of a broad “disengagement crisis” linked to rising crime (Pyrooz et al., 2016; Rosenfeld & Wallman, 2019; Shjarback et al., 2017).

### Police Officers’ Perceptions of Depolicing

Despite the lack of consistent empirical evidence to support a widespread reduction in police activity, many officers remain firmly convinced that such a trend is occurring and numerous studies examining officers’ perceptions have found that law enforcement personnel report a generalized decline in interventions and proactive engagement (Blake & Lafond, 2017; Brown, 2019; Deuchar et al., 2019; Faubert, 2021; Morin et al., 2017; Mourtgos & Adams, 2019; Oliver, 2017, 2019; Phillips, 2020). Officers often use metaphorical language to describe depolicing, framing it as “doing the bare minimum to protect oneself,” “choosing oneself over the mission,” “turning a blind eye,” or “avoiding hot spots” (Béliveau, 2024). While these expressions are somewhat ambiguous, the underlying theme of fear—particularly fear of punitive repercussions—persists in officer narratives. A recurring explanation for the alleged decline centers on the fear of citizen complaints and the possibility of related investigations (Brown, 2019; Foster, 2021; Novak et al., 2003). The growing emphasis on regulatory, ethical, and legal standards has made policing more complex, and many officers feel overwhelmed by what they see as excessive criticism and oversight that affects both their efficiency and their willingness to engage in proactive work (Oliver, 2019). Compounding this frustration, external misconduct oversight systems are often viewed as biased and unjust, composed of civilian investigators who are neither impartial nor reasonable. These perceptions contribute to a deep erosion of trust in oversight mechanisms (Kang & Nalla, 2009; Prenzler, 2015; Wells & Schafer, 2007).

Another key concern among officers is the increasing public exposure of police work, largely due to new technologies that allow for the monitoring and regulation of law enforcement behavior (Goldsmith & McLaughlin, 2022; Søgaard et al., 2022). Officers frequently associate depolicing with the intense media attention surrounding “anti-police” movements such as Black Lives Matter and the viral spread of images that challenge the legitimacy of police practices (Blake & Lafond, 2017; Maguire et al., 2017; Mourtgos et al., 2020). Many feel that this amplified scrutiny is counterproductive and see their disengagement as the result of overly critical and ill-informed public discourse (Cooper, 2020; Gau et al., 2022; Keyes & Keyes, 2022; Mac Donald, 2016). These concerns are further compounded by the belief that civil society lacks a nuanced understanding of police powers and responsibilities (Armony et al., 2023). As a result, officers report that they fear public criticisms, particularly given their potential for judicial consequences (Florence Gauthier & Graziano, 2023).

Most existing research on depolicing focuses on officers’ broad perceptions of social change and increasing oversight, often framing disengagement in abstract terms rather than grounding it in personal experience (Brown, 2019; Edward, 2017; Faubert, 2021; Foster, 2021; Marier & Fridell, 2020; Mourtgos et al., 2020; Nix et al., 2018; Nix & Pickett, 2017; Novak et al., 2003; Oliver, 2017, 2019; Phillips, 2020; Rushin & Edwards, 2017; Warner, 2005; Wolfe & Nix, 2016). Those studies, however, do not examine how individual officers experience public criticism, complaint procedures, or disengagement in their day-to-day realities. While officers often articulate collective anxieties about media attention and legal scrutiny, they seldom describe how these pressures have affected them personally. However, previous research suggests that depolicing does not affect all officers in the same way: while some see it as a widespread and pressing issue, others report little to no change in their professional behavior, highlighting the heterogeneity of experiences within police institutions (Brown, 2019; Faubert, 2021). The present study adopts a focused approach, assessing the validity of officers’ own explanations, particularly the widespread belief that disengagement stems from excessive oversight and fear of citizen complaints. By reconstructing officers’ individual experiences with complaints, media expose, and subsequent procedures, we hope to understand why some police officers report disengaging—or report deeper forms of disengagement—while others affirm they remain professionally committed. Specifically, we examine when and how higher levels of self-reported disengagement occur, and the dynamics that shape divergent testimonies about.

### The Origins of Radical Police Disengagement Narratives

In a previous article (Béliveau et al., 2024), we developed a typology of self-disengagement grounded in officers’ narratives, identifying three distinct levels of disengagement:

1. Engaged officers (36.7% of our sample) reported no significant changes in their approach to policing or public engagement over the course of their careers. While they acknowledged that depolicing exists, they did not see themselves as contributing to it. As one participant stated: “I believe in the phenomenon and can explain its causes, but I am not disengaged and not part of the widespread problem.”
2. Cautiously disengaged officers (47%) adopted a more restrained and selective approach to interventions, characterized by situational reflexivity and apprehension about potential repercussions. While continuing to engage in proactive policing, they tended to describe their practices as more cautious and calculated. Although some perceived this adjustment as a reduction in their overall activity, previous research has shown that such behavioral adaptations can also reflect greater professionalism and stronger adherence to principles of procedural justice (Armony et al., 2023; Black, 1980; Campeau, 2015; Mourtgos et al., 2020; Nix et al., 2024).
3. Radically disengaged officers (16.3%) reported a near-complete withdrawal from interaction with the public and proactive practices. This form of disengagement has been linked to adverse outcomes, including increased police misconduct (Gallup, 2023; Reynolds et al., 2018), rising crime rates (Nix et al., 2024), and a measurable decline in the quality of services provided to communities (Bradford et al., 2009; Braga et al., 2019).

This typology brings to light an often-contradictory relationship between officers’ understanding of depolicing and their actual self-reported behavior: although nearly all participants affirmed the reality and pervasiveness of depolicing, their individual accounts pointed to a much more nuanced picture. The discrepancy between beliefs about the approach being taken by the collective and self-reported behavior underscores the importance of distinguishing between perceived social trends and concrete behavior, particularly as withdrawal, based on our findings, appears to involve only a minority of officers (see also Jobard, 2016; Nix et al., 2024).

The present study examines the origins of radical disengagement narratives, focusing on how officers classified as radically disengaged describe their personal encounters with complaints, media exposure, and subsequent oversight procedures as shaping their level of professional self-reported withdrawal. More broadly, we also draw on the narratives of engaged, cautiously disengaged, and radically disengaged officers to identify how differing experiences contribute to the emergence of the more extreme and socially problematic forms of disengagement discourse.

To examine how officers interpret and narrate their oversight experiences, this study draws on the organizational justice framework (Greenberg, 1987). This framework conceptualizes justice along three interrelated dimensions through which individuals assess the fairness and legitimacy of institutions that monitor, regulate, or publicly scrutinize their work, including complaint mechanisms, police employers, and the media. Procedural justice concerns perceptions of the consistency, neutrality, transparency, and timeliness of decision-making processes. Distributive justice refers to evaluations of outcomes—such as sanctions, exonerations, or public allegations—and their perceived proportionality. Interactional justice addresses the quality of interpersonal treatment throughout these procedures, particularly whether individuals feel treated with respect and dignity (Roberts & Herrington, 2013). Together, these dimensions structure how officers make sense of oversight encounters, frame them as just or unjust, and adjust their professional engagement in response to perceived unfairness (Fridell et al., 2021; Reynolds et al., 2018). The framework thus informs both the interview protocol and the analytic strategy.

## Methods

### Data Source

The data presented in this article are drawn from a broader research project investigating how the mechanisms of police misconduct oversight in Quebec shape perceptions of justice and injustice among complainants, professionals, and police officers. An open invitation to participate was distributed to approximately 15,000 officers across the province, and within four days, nearly 700 had expressed interest in taking part in the study. To ensure a diverse and analytically meaningful qualitative sample, we relied on purposeful sampling (Palinkas et al., 2015). Participants were selected based on multiple criteria: the type of procedure they had experienced (whether internal or external, and whether criminal, ethical, or disciplinary), whether they had been sanctioned, and their organizational affiliation. Additional factors—such as media exposure, the severity and duration of the complaint process as well as sociodemographic factors (e.g., age, years of service, and gender)—were also considered. The aim was not statistical representativeness but theoretical saturation, understood as the identification of a sufficiently varied set of situations that would allow for the emergence of conceptual regularities.

This process yielded a final sample of 59 officers, who took part in formal, semi-structured interviews conducted virtually via Zoom and Microsoft Teams between 2021 and 2022. Two interviewers shared the interviews equally. Each session lasted an average of 1 hour and 57 minutes, with a range of from 50 minutes to 2 hours and 57 minutes. The discussions focused on participants’ experiences with justice, oversight mechanisms and media expose from 2005 to the present, guided by the organizational justice framework (Roberts & Herrington, 2013). All interviews were conducted in French; selected excerpts have been translated into English for the purposes of this article. To ensure consistency across interviews, a semi-structured interview guide was used, and the research team held calibration meetings both prior to and during data collection. Regular debriefings were also conducted to minimize variability in interviewer engagement and interpretation.

Before data collection, several key themes were identified to guide the interviews: events that had triggered the complaints, perceived impact of oversight procedures on participants’ careers and personal lives, and participants’ suggestions for improving oversight practices. Although disengagement was not initially included in the interview guide, it quickly emerged as a central theme. Without interviewer prompting, early participants consistently and emphatically raised the issue, likely reflecting both the personal impact of the complaint process and the broader public debates on the topic in Quebec at the time (notably discussions surrounding Black Lives Matter and increased media attention to police disengagement). From interview 17 onward, disengagement was formally incorporated into the guide. Questions were added about participants’ general perceptions of disengagement, how they experienced or resisted it, and its potential influence on their professional practices. Ultimately, all but two participants raised the subject spontaneously, requiring only occasional follow-up prompts.

### Sample

Participants were active or former police officers from a range of jurisdictions across Quebec, which included Montreal and 24 other regional municipalities or Indigenous communities. Eleven participants were employed by Level 6 services (Sûreté du Québec), 25 by Level 5 (serving populations of 1,000,000 or more), 2 by Level 4 (500,000–999,999), 11 by Level 3 (250,000–499,999), 2 by Level 2 (100,000–249,999), and 8 by Level 1 (under 100,000). Of the 59 officers interviewed, 48 held frontline roles such as patroller, sergeant, lieutenant, community relations officer, or traffic safety officer. The remaining 11 held non-field positions, including director, captain, media relations officer, and detention officer. Eight identified as female and 51 as male;^2^ nearly all identified as white. The average age was 37 years and the mean length of service was 14 years, with the least experienced officer having served for 3 years and the most senior participant being retired. In terms of educational background, everybody had completed the mandatory police training program in Quebec (usually three and a half years long), 14 held a university-level certificate (an undergraduate one-year program), 7 had earned a bachelor’s degree, and 2 had completed graduate-level studies.

### Analytic Strategy

A comprehensive thematic analysis was conducted on all 59 interview transcripts using the qualitative software QDA Miner, incorporating both within-case and cross-case perspectives. In the within-case dimension, each participant’s complaint history was reconstructed, focusing on their experiences with multiple oversight mechanisms, including internal police affairs units^3^ (n = 30), the Commissaire à la déontologie policière (n = 54),^4^ criminal courts (n = 16),^5^ civil courts (n = 12),^6^ the Human Rights Commission (n = 12),^7^ and Serious Incident Investigations Teams (n = 9).^8^ On average, participants reported having faced 6.5 formal complaints over the course of their careers, with every individual having received at least one complaint. Most participants had been investigated by several oversight bodies, both internal and external, and in some cases, multiple procedures occurred simultaneously for the same incident. Several complaints—and their preceding events—attracted extensive media attention, including two fatal shootings that received province-wide coverage.

Coding was conducted inductively, without a predefined grid. Codes were iteratively discussed and refined collectively within the research team, then consolidated into broader themes based on analytical relevance and recurrence. Rigor was ensured through team discussions, triangulation, and constant comparison rather than statistical measures of inter-coder reliability, consistent with an interpretive qualitative approach. A total of 108 inductively generated codes were subsequently organized into two overarching thematic categories: (1) complaint and media experiences, and (2) disengagement. The first category encompassed three dimensions of organizational justice—procedural, interactional, distributive—which were further subdivided into multiple subthemes, depending on the actors involved, the type of procedure, and the specific context of the oversight encounter. Each participant was then classified as Engaged, Cautiously Disengaged, or Radically Disengaged, based on self-reported changes in professional behavior over time, following the typology developed in our previous research (Béliveau et al., 2024). A detailed account of the methodological construction and peer-reviewed validation of this framework is provided in that publication.

The cross-case component of the analysis adopted a comparative, inductive perspective to identify recurring patterns associated with specific forms of oversight experiences and self-reported disengagement, especially in its more radical forms. Both inter-case comparisons (across participants) and intra-case comparisons (within participants across different oversight bodies) were conducted. This approach clarified not only which oversight dynamics were linked to particular disengagement narratives, but also why certain experiences were more likely to generate extreme reactions. Consistent with the inductive manner in which disengagement emerged during fieldwork, the aim was not to test a predefined hypothesis but to construct an interpretive framework capable of accounting for the processes shaping disengagement narratives. In this perspective, disengagement is treated not as a quantifiable outcome but as a relational process shaped by lived experiences of oversight, media exposure, and complaint procedures (see Søgaard et al., 2022 for a comparable analytic strategy). For analytical depth, the findings presented in this article focus primarily on experiences reported by Radically Disengaged Officers, as these cases most clearly illustrate the organizational dynamics underlying problematic forms of withdrawal.

## Results

A first general observation from our results is that the level of disengagement seems to be directly linked to officers’ perceived validity of the oversight experience (perceived as positive versus negative) as well as the type of oversight procedure (internal versus external; see Table 1).

**Table 1.** Levels of disengagement by Complaint Experience^9^ and Oversight System

		Overall complaint experience	
		Perceived positive experience	Perceived negative experience
Oversight system	External organizations	1 neutral or positive impacts – includes most engaged officers and some cautiously disengaged officers	2 minor negative impacts (mainly stress) – includes most cautiously disengaged officers and some engaged officers
	Internal affairs	3 neutral or positive impacts – includes most engaged officers and some cautiously disengaged officers	4 highly negative impacts – includes all, and almost exclusively, radically disengaged officers

Radical disengagement narratives were not associated with media exposure or external complaints but were almost exclusively linked to experiences with internal affairs, which represent the type of oversight procedure that attracted the least media attention. All officers in this group had taken part in such procedures and perceived them as profoundly unjust. While not all those who faced internal discipline reported radical disengagement, those who did invariably described these processes as punitive and linked to changes in their attitudes and professional behavior. This sense of injustice stemmed largely from how internal complaints were managed, with many officers viewing internal affairs procedures as having a more severe and damaging effect on their career than external oversight—particularly because the process originated within their own organization. As one officer explained, “The internal responses are rougher than those that come from dealings with citizens. A complaint from the Commissaire à la déontologie policière—that’s not really what scares me versus what the internal service is going to do to me. How it will turn my work environment into a kind of nightmare.” (#14)

Our within-case and cross-case analyses revealed that the design of internal disciplinary system procedures—particularly the way complaints are addressed and how the organization responds to different types of incidents—helps explain why these experiences, more than others, are associated with radical disengagement narratives. In the following sections, we examine in detail the three interconnected organizational dynamics of injustice that underlie this phenomenon: (1) the officer’s inability to externalize blame due to the internal origin of the complaint; (2) the stigmatizing effect of precautionary administrative measures, such as suspension or reassignment; and (3) the perceived betrayal of core occupational values when the discipline targets what are seen as morally justified actions. Together, these dynamics illuminate how internal disciplinary encounters can fracture officers’ organizational loyalty and foster extreme, problematic, and socially undesirable forms of disengagement discourse.

### Internal Investigations: Perceived Betrayal and the Inability to Externalize Blame

Regardless of the nature or outcome of the charges—and whether the oversight process was internal or external—most participants felt they were not responsible for the allegations brought against them (Herzog, 2001; Mulone & Boivin, 2017). Officers commonly described themselves as exemplary and blameless, characterizing the investigations as unnecessary and often labeling the complaints as “frivolous” or “malicious”—terms that recurred frequently throughout the interviews. Despite their personal frustrations, most participants still recognized the importance of oversight in cases of genuinely deviant behavior by fellow officers. However, they attributed complaints against themselves as the result of minor errors in judgment, bad faith by complainants, or flaws in the complaint-handling system or in society at large. As one officer put it, “The complaint would’ve never been upheld if someone had actually reviewed it properly” (#40). Another remarked, “Given the circumstances, my take is that we were made an example—because we’re caught in this post-George Floyd social wave … We filed a complaint against the judge because her ruling contained a clear and significant error. Honestly, the whole decision felt heavily influenced by subjectivity. And the judge was a member of a minority group …”

It is reasonable to assume that, while some complaints may be unfounded or exaggerated, a significant subset reflect actual misconduct—particularly since 40% of the sample had been formally found guilty of at least one allegation following thorough investigations. Yet, nearly all participants categorically denied the accusations against them. At least half of the deniers appeared both sincere and credible: their narratives often cast doubt on the integrity of the complaint process and aroused the sympathy of the interviewer. However, a significant minority of officers seemed either unwilling or unable to acknowledge any wrongdoing. Their firm denials seemed implausible and, in some cases, undermined their credibility. For example, some participants justified actions that were explicitly illegal, arguing that these acts had been unfairly targeted by internal disciplinary mechanisms. For instance, Participant #15 expressed resentment over being investigated for driving a personally owned vehicle that had been illegally modified, while Participant #06 described his frustration over being arrested for driving over 200 km/h on a motorcycle—an act that violates not only disciplinary codes but also criminal law. Similarly, Participant #17 was outraged at having been treated “like a thug” when he and a colleague were arrested by two officers for smoking cannabis (prior to its legalization in Canada) while off-duty. The situation escalated into a verbal and physical confrontation. Although both men were ultimately found guilty and sanctioned through internal disciplinary processes, they continued to see the other officers as the true wrongdoers and insist that they had been unjustly arrested, despite openly admitting to having committed the offense in question.

In this second type of narrative, a close reading of officer testimony reveals internal contradictions that cast doubt on the legitimacy of the narration of the account. Such narratives align with theories of moral disengagement and techniques of neutralization that explain how individuals deflect personal responsibility by shifting blame to external sources. Actors, whether delinquents or police officers, often use such strategies to retroactively justify actions they might otherwise recognize as inappropriate or wrongful. These mechanisms allow individuals to reinterpret their behavior using situational justifications or circumstantial logic (Bandura, 1990; Cromwell & Thurman, 2003; Sykes & Matza, 1957). In our study, the tendency to deny responsibility was widespread—the vast majority of interviewees firmly believing that the police intervention which led to a complaint was professional and lawful, even among officers who did not report being radically disengaged. This raises a key question: if such rationalization is common across the board, what differentiates the officers who escalate to more extreme and problematic forms of disengagement narratives?

One significant difference is the way internal affairs investigations are initiated. Investigations initiated by external oversight agencies are typically the result of public complaints or based on predefined criteria related to serious incidents, meaning that officers are usually able to predict that an investigation will take place and what it will involve, while internal disciplinary investigations often originate from within the organization itself and are frequently initiated by the employer when officers are suspected of violating internal codes of conduct or the criminal code, even in the absence of any external complaint. Radically disengaged officers were subjected more frequently to employer-initiated investigations than to investigations triggered by civilian complaints, a distinction that was central to shaping their sense of injustice. For many of these officers, the absence of a civilian complaint meant that responsibility for the complaint could not be externalized, leaving as its source either their own wrongdoing or their own organization—a dilemma that many found deeply disorienting. The inability to deflect blame externally became a source of moral tension.

“I've had several ethics complaints in the past: always on unfounded matters. But I would say the biggest thing I’ve experienced was an internal complaint I received ... Actually, it wasn’t really a complaint, but it was difficult, complicated, and all messed up. I think, just that, we could talk about it for four hours.” (#32)

By contrast, participants who had negative experiences with external oversight bodies, regardless of their actual guilt, were typically able to rationalize those experiences by blaming the complainants or criticizing the investigative process.

“That complaint? It was clearly about revenge — no doubt about it. Every single ethics complaint I’ve ever received, without exception, was rooted in retaliation. These were people who had been arrested, ticketed, or sanctioned … and they used the complaint process to get back at me. So, if you’re asking whether I trust the ethics system? Not for a second. No one ever looks at whether the mistake was made in good faith. It’s all about numbers — about showing the public: 'Look, we take your complaints seriously.'”

External attribution neutralized their sense of injustice, in sharp contrast to the unresolved frustration generated by internally initiated investigations. Beyond the psychological discomfort of the moral ambiguity of internal investigations—whether to acknowledge fault or blame the organization—such investigations were also experienced as a betrayal. Internal proceedings initiated by fellow officers violated expectations of solidarity within the police force.

“In the policing community, if someone tells you, 'I work in Internal Affairs,' I’m done talking to them. I don’t want anything to do with that person. Internal Affairs investigates cops. Why would I want to be friends with someone who might stab me in the back one day? And that’s what ethics investigators are too — people who chose to go after cops, to take down fellow officers. That’s their job: punishing cops for ridiculous things.” (#35)

The authority given internal affairs to investigate their colleagues conflicts with the ethos of loyalty traditionally seen as foundational to police culture—an implicit code of mutual support (“we stand by each other”) (Campeau, 2015; Reiner, 1992). Officers who are strongly supportive of these cultural norms are, statistically, more likely to become the subject of citizen complaints (Terrill & Ingram, 2016). In summary, this first dynamic of injustice arises from two interconnected factors: the perceived betrayal of internal solidarity and the inability to rationalize accusations that originate from within their own institution.

### Precautionary Measures: Stigmatization, Isolation, and Professional Alienation

In Quebec, when there is evidence to support serious allegations against officers, such as criminal charges, police disciplinary departments may impose a range of “precautionary measures” during the investigation process. Unlike external oversight bodies, internal police employers have broad discretionary powers during active proceedings, including disarming officers, advising them of their rights, issuing gag orders, reassigning duties, entering private residences, and authorizing surveillance, seizures, or wiretaps. Officers may also be suspended or removed from active duty. These measures are routinely enforced regardless of whether the initial complaint originated internally or through an external agency. For those in our sample, such precautionary measures were most commonly associated with internal procedures and far less frequently observed in the context of external oversight. They appear to be structurally linked to internal disciplinary departments within police organizations. Radically disengaged officers frequently reported having been subjected to these precautionary measures—often more than once. Their testimonies powerfully convey the sense of exclusion, stigmatization, and professional humiliation such procedures can induce. For example:

“It took almost a month to the day before I was removed from duty. Basically, it was simple. I arrived one day at the station, and they told me: ‘Grab your stuff, you're not coming in here anymore. You’re going to headquarters, and we’ll call you at some point.’ So, I went to headquarters as punishment. I spent a year there. It took a year to the day before I got a response from the District Attorney saying I had no charges. Since the criminal case was dropped, I was able to return to duty, but I still have ongoing procedures with the Human Rights Commission and ethics.” (#28)

Another officer recounted a more invasive and psychologically distressing experience:

“They opened an internal investigation on my colleague, who was seeing erotic masseuses and prostitutes. He ended up under wiretap surveillance. At some point, while he was being recorded, he was talking to me about all kinds of things. Internal Affairs had set up what we call a provocation plan, related to a massage parlor he had visited. Long story short, they ended up arresting him. As for me, Internal Affairs brought me in as a respondent. I was forced to give a disciplinary statement. They even played fake wiretap recordings — completely fabricated — trying to push me into confessing something. They also threatened me regarding the lieutenant-detective promotion I was waiting on at the time. Honestly, it was probably the most botched investigation I’ve ever seen in my life. It was illegal, just a mess of distorted facts. That’s how I found out my colleague was being wiretapped — and so was I, just because I’d talked to him. I could’ve said something totally meaningless, completely out of context, and gotten charged for something I didn’t even do. It would’ve been devastating. I don’t think I’ve ever felt that disengaged in my entire career. Never. Never, never, never, never, never… Never!” (#07)

In contrast, participants from cluster 3 (see Table 1)—mainly engaged officers—often highlighted the *absence* of precautionary measures during disciplinary procedures as a key factor in maintaining their trust in the system and their professional commitment. For some, simply being allowed to continue working was a protective factor against disengagement:

“The other thing that helped me get through the disciplinary process was that they let me stay on duty. I wasn’t sent home. Honestly, if I’d been suspended and forced to stay home on top of everything else, I think I would’ve ended up quitting. Not knowing where my case was headed, not knowing what it would be like coming back … At least by staying at the station, I could talk to other colleagues. We’d go over what happened, talk about how outrageous the inspector’s behavior was, and how internal affairs were blowing things way out of proportion. That’s what helped me — staying in the service while the whole thing played out. Being able to talk to people, hear the chatter in the hallways. And sometimes, I could set the record straight. When I heard certain things going around, I’d step in and say: “Hey, hold up! Don’t go spinning a wild story — you have no idea what you’re talking about!” (#01)

For radically disengaged officers, precautionary measures were not seen as neutral procedural safeguards but as unjust punishments imposed in the absence of any formal conviction. Repeatedly describing the result as having been treated “like a criminal”—a recurrent and emotionally charged phrase—these officers experienced precautionary measures as both stigmatizing and deeply alienating. Among the various actions taken, those that resulted in isolation—removal from duty, reassignment, or suspension—were most often cited as particularly painful, interpreted less as precautionary protocols than as symbolic forms of ostracism. Participants emphasized the immense value police culture places on professional reputation, describing it as a fragile but vital form of capital. In an environment often characterized by informal gossip, internal rivalries, and backchannel communication, reputation was seen not only as a marker of credibility but also as the currency of belonging and advancement. In this context, so-called “precautionary” actions heightened visibility at precisely the moment officers most wished to remain unseen. While peers might distance themselves from those perceived as “bad apples”, a competing impulse toward maintaining solidarity could also emerge—leading to rumors over which the targeted officer had little control. Once removed from their peer group, officers described losing the ability to shape their own narrative, an erosion of agency over professional identity that fostered deep anxiety. Within the policing world, being talked about was not merely uncomfortable but consequential: reputation shaped informal hierarchies, influenced trust among colleagues, and often determined access to desirable roles or promotions. The fear that precautionary measures would result in “losing face” and returning to the workplace as a marginal figure was especially dreaded.

“Once you get pulled into that sausage machine — internal affairs, special investigations — you enter a space where everyone looks at you like, “Yeah, I knew it, he was dirty. I just knew it.” We’re judged harshly by our peers — and we judge ourselves just as much. We become our own worst enemies. The guilt, the shame, the doubt … it eats away at you from the inside. That look from your peers — from people who were family — I felt it. That discomfort was real. And it only got worse because I was told not to talk about the incident. I had to keep quiet so I wouldn’t interfere with the investigation. I couldn’t reassure anyone, couldn’t share any details. And that just fed the rumor mill. I lost a lot of friends because of the whispers, the gossip. I had to transfer to a different precinct. Now, when we pass each other, we barely even say hi. They look at me with contempt!” (#06)

“After being suspended for a year at home — with my partner [who’s also a police officer] on paid leave during the investigation — coming back was tough. Because of the isolation, people had kind of gotten used to not talking to us. We felt like outsiders, like we’d been pushed to the margins. Especially my partner. Later on, she applied for several investigative positions, but she never made it past the interviews. And yet, every time I have a question about investigations, she’s the one I turn to. She’s sharp, she knows her stuff, she applied more than once … But in this line of work? Investigative jobs — people talk.” (#25)

Radical disengagement discourses were triggered not only by the accusation itself but also by the informal social penalties that followed. In contrast to external complaints (cluster 1 and 2, see Table 1)—which typically stemmed from interactions between police and the public, such as communication breakdowns or minor service issues—internal disciplinary cases were often rooted in interpersonal tensions, workplace conflicts, and “turf wars” among colleagues, typically centered on power dynamics, status, or hierarchy. This internal dimension amplified the fear of “losing face,” especially given that such disputes occur between peers and can escalate into formal disciplinary action—sometimes even criminal investigations.

Finally, it is worth examining the role of sanctions in shaping officers’ levels of disengagement. In our sample, internal disciplinary procedures were more likely than external investigations to result in formal sanctions.^10^ This observation might suggest that the outcomes of such internal investigations—particularly being found guilty—contribute directly to disengagement. However, our interviews revealed a more complex reality: in most cases, the sense of injustice emerged long before any verdict was rendered, rooted in the investigative process itself. For radically disengaged officers, the injury was not limited to the sanction per se but involved the experience of being isolated, stigmatized, and symbolically repositioned as an offender—despite occupying the role of enforcing the law. This reversal—being cast in the role of the accused—was deeply unsettling for their moral identity and organizational belonging. Participants described this rupture as involving three interrelated dimensions: Social isolation, resulting from reassignment and exclusion from their peer group; Professional isolation, marked by removal from operational duties; and Cultural isolation, characterized by suspension of the symbolic and moral status associated with being a police officer (Campeau, 2015; Reiner, 1992).^11^ According to theories of procedural justice, seeing the process as fair—not the avoidance of punishment—is the strongest predictor of feelings of legitimacy and institutional trust (Prenzler, 2015). Yet, the way internal disciplinary systems are structured—notably their ability to initiate investigations in the absence of any external complaint and their use of precautionary measures—often undermines this perception. But does this mean that internal disciplinary mechanisms are intrinsically flawed or counterproductive? To explore this question further, we now turn to a third and final overarching dynamic of perceived injustice.

### Higher Loyalties and Value Conflicts: Noble Cause Corruption and Organizational Mistrust

In addition to denying responsibility, many radically disengaged participants also invoked higher loyalties as a neutralization technique (Cromwell & Thurman, 2003). These officers frequently justified their actions by claiming that they aligned with core professional values, portraying their conduct—even when acknowledged to be deviant—as morally justified and rooted in a greater sense of duty. This phenomenon is commonly referred to as “noble cause corruption”, in which misconduct is framed as serving a greater good (Caldero et al., 2018). A striking example of this dynamic came from Officer #14, who had been the subject of multiple employer-initiated investigations. In an investigation following his refusal to obey a direct order to abandon a high-speed pursuit he insisted, “I couldn’t let that criminal get away.” Another case involved the excessive use of force against a verbally disrespectful civilian, and a third stemmed from an incident in which he struck an intoxicated detainee. Reflecting on his actions, he explained: “I embody a figure of authority; I couldn’t let myself be disrespected … Older officers did much worse … When you act in good faith, you’re sanctioned.”

Allegations against radically disengaged officers that originated internally were not only more serious and more likely to be rooted in peer conflict than external complaints but also shared a striking internal consistency: the incidents reported by officers in our sample were often highly specific and described using similar emotional and moral framing. Many officers expressed great confusion over why they were being investigated by their own organization, especially when they believed their actions had been aimed at protecting the public. Their appeals to a higher loyalty were frequently interwoven with narratives of proactive engagement in crime suppression. Many of these officers had been heavily involved in active policing, often exceeding the performance of their peers. The disciplinary response from their employer undermined their sense of mission. Participant #32 offered a particularly revealing account:

“There are officers who arrest one or two people a year. In 2018, my partner and I made 210 arrests between the two of us. Yes, we stood out, but we weren’t arresting small-time shoplifters or bike thieves. It was all possession with intent to traffic, possession of weapons, obstruction of justice because the guy gave us a false identity. It was always related to serious criminal acts ... As soon as my boss started targeting me and even put me under observation without telling me, I doubled my caution, and we started working less ... I didn't know that arresting too many criminals was a problem. Now I understand; my organization doesn’t like that. My organization is rotten from the inside.”

This narrative illustrates how what is seen as organizational mistrust leading to scrutiny can erode officers’ engagement—especially when they believe their actions reflect commitment rather than misconduct. While Participant #32 admitted that his methods were “deviant” compared to institutional norms, he still considered his work exemplary. For him, 210 arrests in a single year was a noble and necessary contribution to public safety. Rather than seeing their conduct as problematic, radically disengaged officers often criticized their institution—and society at large—for being complacent, inefficient, or excessively lenient. They considered aggressive crime-fighting to be a moral imperative for police officers and disciplinary interventions to be a betrayal of that imperative (Cobbina-Dungy & Jones-Brown, 2023), reflecting a deep disconnect between their internalized sense of moral duty and the behavioral expectations of their organization. Disciplinary oversight—especially when it involved officers who saw themselves as morally driven—triggered withdrawal not only from fieldwork but also from the organization’s normative framework. Officers’ disengagement narratives were rooted in a conflict between values: between what they believed policing *ought* to be and what their institution demanded of them.

“I’m currently working in a neighborhood known for its high crime rate. Street gangs are very present — more and more so, actually. But every time my partner and I get a complaint, it’s getting harder to justify staying proactive. The reality is, I’ve got colleagues who write one or two tickets a day, drive around aimlessly, and just respond to the calls they’re dispatched to. Yet they get paid the same salary and receive the same performance evaluation as I do. I’m not saying this to criticize them — everyone’s different, and we can’t expect the same level of output from everyone, and that’s fine. But the truth is, I carry out more interventions than they do, which means I’m more at risk of things escalating. I’m more likely to end up using force. And the more force I use, the greater the risk of injuring someone or triggering a serious incident, unfortunately. The worst part is, in this police service, the more use-of-force reports you write, the more you’re watched by your employer. I keep talking about use of force, but just to give you an idea: in a single year, I wrote about three-quarters of all the use-of-force reports in the service”. (#44)

“I used to be deeply involved in investigations. I had sources who gave me solid intel. I was working on surveillance files, stakeouts — you name it. But all of that is over now … Proactive policing isn’t valued anymore in performance metrics. So why would I put myself in a situation where my police service won’t even have my back? I’ve been through it … The service never backs you up. That internal investigation took away my sense of initiative as a cop. I’m in my 17th year now, with 8 more to go. By 25 years in — maybe even sooner — I’ll retire and move on to a different career, one where I’ll actually be seen as a person”. (#06)

Police interventions that led to internal investigations—such as high-speed chases or specific arrest patterns—were seen by many officers as a direct challenge to their role and identity as police professionals. Their discussion of these experiences reflected not only individual frustration but also deeper cultural sources of perceived injustice—particularly a sense that institutional support for crime fighting, as a core mission of policing, was lacking.

## Reframing Depolicing as an Organizational Phenomenon

This study set out to explore the roots of police disengagement narratives. When asked directly about the reasons behind their self-reported behavioral changes associated with what they call depolicing, participants consistently cited external factors—notably civilian oversight systems and media scrutiny—as primary causes, echoing findings from previous studies (Brown, 2019; Gau et al., 2022; Marier & Fridell, 2020; Mourtgos et al., 2020; Nix et al., 2018; Nix & Pickett, 2017; Novak et al., 2003; Oliver, 2017; Rushin & Edwards, 2017; Wolfe & Nix, 2016). No one in our sample explicitly linked their disengagement to internal disciplinary procedures. However, cross-case analysis comparing the depolicing typology developed in our prior work (Béliveau et al., 2024) with officers’ personal oversight experiences showed that radical disengagement discourses—the highest level of disengagement identified in our sample and the only socially problematic form—were most closely associated with a sense of injustice related to actions by their employer, particularly in cases involving employer-initiated investigations and the use of precautionary measures or sanctions following proactive crime-control interventions. Although these three actions were not always experienced together, participants who reported them in combination described a recognizable pattern of radical disengagement narratives Figure 1.

**Figure 1.**
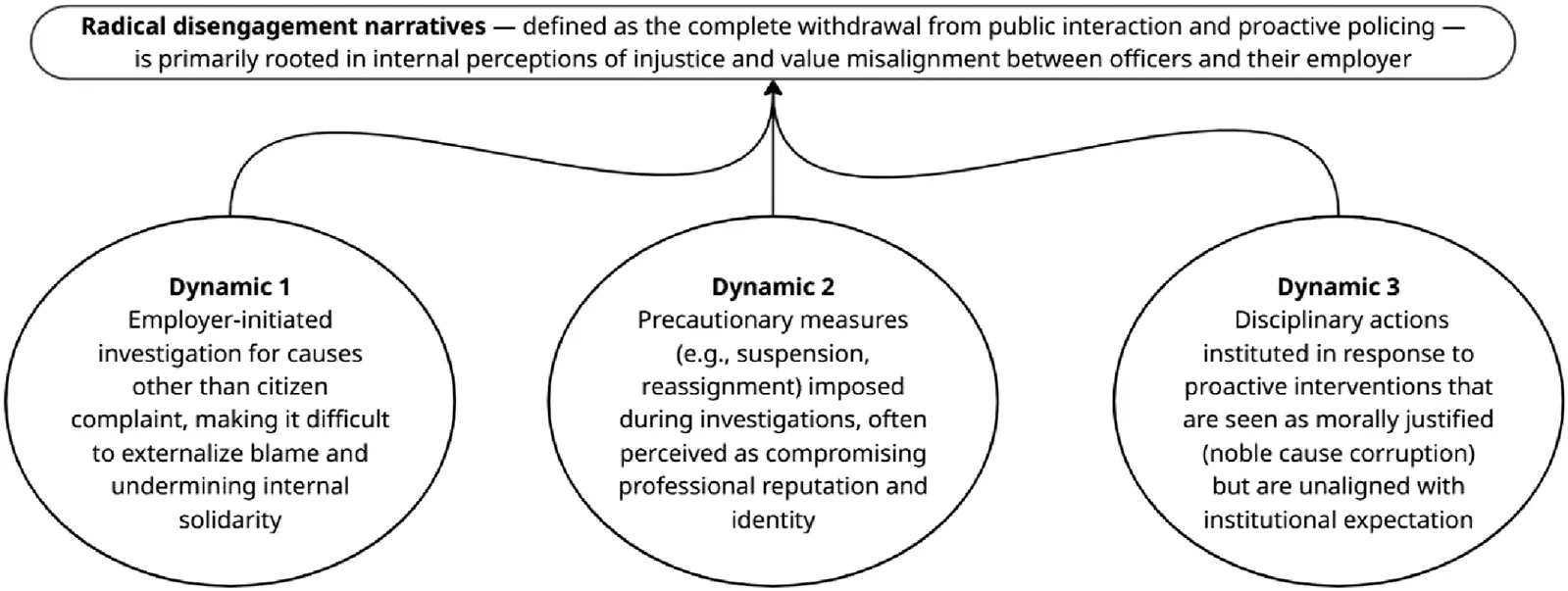
Three organizational dynamics underlying radical police disengagement narratives

Monjardet (1996) described disengagement rhetoric as the result of having to act in ways that contradict an officer’s understanding of the police mission—a process he termed *disengagement by obligation*. Our findings suggest a complementary mechanism: *disengagement by deprivation*—the result of frustration experienced by officers who feel they have been stripped of the authority to perform tasks they once viewed as legitimate, necessary, and meaningful. When internal controls increasingly constrained their practices, these officers experienced a loss of autonomy in shaping their role within the crime-control mission, triggering a progressive withdrawal from core policing duties. However, this process might also be interpreted as a specific variant of *disengagement by obligation*, particularly in the case of officers whose professional ethos was especially narrow—that is, those whose sense of the meaning of their work was based almost exclusively on enforcement-focused activities such as investigations, arrests, or restraining individuals felt to be threatening. These officers often lacked the capacity—or willingness—to redefine their professional identity or to assign value to other dimensions of police work, seeing them not only as not integral to their mission but as bureaucratic burdens lacking moral or professional significance. Erosion of agency over traditional practices proved particularly disorienting for such individuals, even though more than 83% of police interventions today involve non-criminal situations, such as conflict mediation or community assistance (Willis & Mastrofski, 2014). In contrast, officers categorized as engaged or cautiously disengaged (clusters 1, 2, and 3 in Table 1) typically had a broader view of their professional ethos. While still committed to crime control, they also expressed strong investment in social intervention, public engagement, service quality, and extrajudicial approaches. As frontline practitioners, they showed sustained interest in addressing psychosocial issues and responding to service calls. Their vision of “good policing” emphasized presence, commitment, and community integration, consistent with the principles of procedural justice and community-Oriented Policing (Goldstein, 1987; Sunshine & Tyler, 2003). Identifying themselves as generalists, rather than solely crime-fighters, they appeared better equipped to remain engaged, even when confronted with oversight, complaints, or organizational friction. Thus, while our findings reinforce Monjardet’s theoretical framework, they also extend it, suggesting that officers with a rigid, enforcement-centered conception of police work whose professional trajectory has been disrupted by internal disciplinary processes may be particularly vulnerable to radical disengagement discourses.

Some scholars have suggested that discussion of police disengagement can, at times, serve strategic purposes, for instance by being mobilized in negotiations over working conditions (Chanin & Sheats, 2018), resisting organizational or societal demands (Brehm & Gates, 1999; Oliver, 2019), or even evading accountability, particularly in contexts involving race and discrimination (Black, 1980; Cooper, 2002, 2003, 2020; Keyes & Keyes, 2022). From this perspective, disengagement rhetoric is not merely a passive symptom of occupational frustration but also a political resource—a way for officers to assert control or express dissent.

From another perspective, disengagement narratives can be understood as arising from dissonance between an officer’s idealized expectations of the role and the complex realities encountered in the field (Alain, 2011). Among radically disengaged officers, this disconnect is amplified by the belief that their employer is repudiating practices once considered essential to crime control (Cobbina-Dungy & Jones-Brown, 2023). As a result, officers who formerly took pride in proactive enforcement now adapt only reluctantly to new institutional norms—not out of conviction but to protect their careers and reputations. In doing so, they are forced to abandon what they once viewed as their core professional mission.

Depolicing, therefore, is connected not only to individual dissatisfaction but also to frustration with police structure and expectations (Edward, 2017; Hoffman et al., 2023), particularly the organization and governance of contemporary police institutions. Misalignment between individual values and institutional norms has been shown to significantly undermine professional engagement and has been associated with compassion fatigue, burnout (Andersen & Papazoglou, 2015; Correia et al., 2023), psychological distress (Shane, 2010; Violanti & Aron, 1993), and reduced job satisfaction (Crow et al., 2012). Dealing with depolicing therefore requires determining whether the radically disengaged officers in our sample, who defined their identity primarily in relation to crime-fighting, were simply unable or unwilling to adapt to the evolving demands of frontline policing or if the frameworks within which police officers are managed, evaluated, and supported should be reconsidered (Chan, 1996; Gau et al., 2012).

## Limitations, Institutional and Research Implications

This study has several limitations, but they open avenues for practical reflection and future research. First, selection and social desirability biases must be acknowledged. The original project was not designed to measure depolicing directly, and participation may have attracted officers with stronger feelings of injustice or dissatisfaction. The average of 6.5 complaints per officer reported in our sample is unlikely to represent the Quebec police population at large (Terrill & Ingram, 2016). Moreover, officers under investigation may have had incentives to deny wrongdoing, frame themselves as victims, or portray reduced proactivity as a form of protest. Such dynamics could have influenced the narratives collected. That said, our aim was not to quantify depolicing behaviorally but to examine how officers narrate disengagement in relation to oversight experiences. From this perspective, the potential overrepresentation of radically disengaged officers is analytically valuable: it provides rare access to a phenomenon that may be numerically marginal but socially consequential. While most officers remain engaged, focusing on those reporting the strongest sense of injustice highlights the dynamics most likely to produce problematic forms of withdrawal.

Second, although narratives cannot be equated with observable behavioral change, organizational research consistently shows that perceptions of procedural and distributive injustice increase the risk of disengagement, deviance, or reduced adherence to institutional goals. Our findings suggest that, for radically disengaged officers, the relationship with leadership was not simply strained but fundamentally fractured. In policing, such perceptions can undermine procedural justice in encounters with the public, sometimes escalating to misconduct, abuse of authority, or excessive use of force (Bradford & Quinton, 2014; Correia et al., 2023; Fridell et al., 2021; Lawson et al., 2022; Reynolds et al., 2018; Roberts & Herrington, 2013; Tankebe, 2013). Radical disengagement narratives should thus be seen not only as coping strategies but also as potential precursors of behaviorally significant outcomes. Self-reported withdrawal from both public interaction and proactive fieldwork may erode institutional legitimacy and contribute to declining public trust and social cohesion (Bradford et al., 2009; Gau et al., 2012; Roberts & Herrington, 2013; Sunshine & Tyler, 2003). On a macro level, citizens who trust the police are more likely to cooperate, accept institutional authority, and support legitimacy; on a micro level, negative encounters weigh disproportionately, with one adverse experience undermining trust far more than multiple positive ones can restore it (Bolger et al., 2021; Bradford, 2011; Skogan, 2006). While public attention often focuses on external oversight mechanisms, our data suggest that internal affairs units occupy a unique and understudied position. By virtue of their organizational proximity, they are uniquely placed to monitor, investigate, and address misconduct—as is the case in other professional fields (Brehm & Gates, 1999; Gallup, 2023). Internal affairs may function as moral frontiers, mediating between officers’ expectations of policing and the public’s evolving demands. To fulfill this role effectively, however, they must be perceived as reinforcing professional integrity and institutional trust rather than as purely punitive. When experienced as unfair, internal disciplinary processes may transform ‘deviance’ into new forms of ‘deviance’—what we call ‘radical depolicing’ or organizational mistrust.

This study is exploratory and qualitative. It does not claim causal inference, nor does it suggest that disciplinary processes automatically lead to disengagement. Instead, it highlights narrative linkages between oversight experiences and self-reported withdrawal. It is therefore neither prescriptive nor reformist. Future research should:

• Use quantitative and behavioral data to validate or nuance our findings, measure the prevalence of engagement profiles (engaged, cautiously disengaged, radically disengaged), and assess their causes, organizational dynamics, and societal impacts.
• Control for additional factors not examined here (e.g., officer demographics, burnout, job satisfaction, policy changes, scandals, public trust trends).
• Explore the interaction between internal justice perceptions and external accountability mechanisms, and their joint effects on professional identity and public legitimacy.
• Investigate targeted reforms to address or reintegrate radically disengaged officers, and more broadly those who perceive organizational injustice, in order to mitigate the risks posed by cynicism, withdrawal, or deviant behaviors.

## Data Availability

Due to ethical restrictions and confidentiality agreements approved by the Université de Montréal Research Ethics Board, the full transcripts of the semi-structured interviews conducted with police officers, as well as related supporting materials, are not publicly available*.

## Notes

1. For reading in the media on the subject, see Abt, T. (2022, November 25). “The U.S. Knows How to Reduce Crime.” Wall Street Journal. https://www.wsj.com/articles/the-u-s-knows-how-to-reduce-crime-11669391854; Dewan, S. (2017, March 29). “Deconstructing the ‘Ferguson Effect’”. The New York Times. https://www.nytimes.com/interactive/2017/us/politics/ferguson-effect.html; Fox, J. (2023, May 30). “Fighting Crime Requires More Police and Less Prosecution”. Washington Post. https://www.bloomberg.com/opinion/articles/2023-05-30/fighting-crime-requires-more-police-and-less-prosecution; Martinez, M. (2015). “Baltimore sees deadliest month in 15 years”. CNN. https://www.cnn.com/2015/05/26/us/baltimore-deadliest-month-violence-since-1999/index.html; CBS News. (2017, January 11). “Police officers more reluctant to use force, make stops, Pew survey finds”. https://www.cbsnews.com/news/police-officers-reluctant-use-force-make-stops-pew-survey-finds/.

2. One participant identified himself as a trans man.

3. Internal police affairs refer to units within the police organization responsible for conducting internal investigations and disciplinary procedures involving employees (police officers).

4. The Commissaire à la déontologie policière is an external civilian oversight body mandated to investigate and monitor breaches of professional standards and the code of ethics.

5. Criminal allegations are dealt with through the conventional criminal justice system, in which officers may be prosecuted for alleged violations of the Criminal Code.

6. Civil cases are dealt with within the civil justice system and typically involve lawsuits over damages arising from police actions.

7. The Commission des droits de la personne et des droits de la jeunesse is an external civilian oversight body that investigates police conduct from a human rights perspective, including issues related to discrimination.

8. The Bureau des enquêtes indépendantes is a hybrid external oversight agency composed of both civilian investigators and police experts. It is mandated to investigate serious incidents involving police officers, including those resulting in death or serious injury.

9. During the interviews, officers provided approximately 180 in-depth accounts of complaint experiences. Of these, nearly one-third involved internal complaints, while just over two-thirds involved external complaints. For both internal and external complaints, about half of the officers described their experience as being perceived positively, focusing on personal benefits (notably learning opportunities), while the other half described the experience as being perceived negatively, associated with impacts they saw as unconstructive.

10. Within the sample, two officers were sanctioned by the Commission des droits de la personne et des droits de la jeunesse (2 out of 12, or 16.7%), seven by the Commissaire à la déontologie policière (7 out of 54, or 13%), and twelve by their employer—some on multiple occasions (12 out of 30, or 40%).

11. For several participants, police work occupied a central—if not foundational—role in shaping both their identity and everyday personal life.
